# Differential regulation of *myc* homologs by Wnt/β‐Catenin signaling in the early metazoan *Hydra*

**DOI:** 10.1111/febs.14812

**Published:** 2019-03-26

**Authors:** Markus Hartl, Stella Glasauer, Sabine Gufler, Andrea Raffeiner, Kane Puglisi, Kathrin Breuker, Klaus Bister, Bert Hobmayer

**Affiliations:** ^1^ Institute of Biochemistry University of Innsbruck Austria; ^2^ Center for Molecular Biosciences Innsbruck (CMBI) University of Innsbruck Austria; ^3^ Institute of Zoology University of Innsbruck Austria; ^4^ Institute of Organic Chemistry University of Innsbruck Austria; ^5^ Present address: Department of Molecular, Cellular, and Developmental Biology University of California Santa Barbara CA USA

**Keywords:** cnidarian, development, gene regulation, oncogene, signal transduction

## Abstract

The c‐Myc protein is a transcription factor with oncogenic potential controlling fundamental cellular processes. Homologs of the human c‐*myc* protooncogene have been identified in the early diploblastic cnidarian *Hydra* (*myc1*,* myc2*). The ancestral Myc1 and Myc2 proteins display the principal design and biochemical properties of their vertebrate derivatives, suggesting that important Myc functions arose very early in metazoan evolution. c‐Myc is part of a transcription factor network regulated by several upstream pathways implicated in oncogenesis and development. One of these signaling cascades is the Wnt/β‐Catenin pathway driving cell differentiation and developmental patterning, but also tumorigenic processes including aberrant transcriptional activation of c‐*myc* in several human cancers. Here, we show that genetic or pharmacological stimulation of Wnt/β‐Catenin signaling in *Hydra* is accompanied by specific downregulation of *myc1* at mRNA and protein levels. The *myc1* and *myc2* promoter regions contain consensus binding sites for the transcription factor Tcf, and *Hydra* Tcf binds to the regulatory regions of both promoters. The *myc1* promoter is also specifically repressed in the presence of ectopic *Hydra* β‐Catenin/Tcf in avian cell culture. We propose that *Hydra myc1* is a negative Wnt signaling target, in contrast to vertebrate c‐*myc*, which is one of the best studied genes activated by this pathway. On the contrary, *myc2* is not suppressed by ectopic β‐Catenin in *Hydra* and presumably represents the structural and functional c‐*myc* ortholog. Our data implicate that the connection between β‐Catenin‐mediated signaling and *myc1* and *myc2* gene regulation is an ancestral metazoan feature. Its impact on decision making in *Hydra* interstitial stem cells is discussed.

**Abbreviations**
Alp: alsterpaullone
bHLHZip: basic region/helix‐loop‐helix/leucine zipper
CEF: chicken embryo fibroblasts
ChIP: chromatin immunoprecipitation
ESI‐MS: electrospray ionization mass spectrometry
GFP: green fluorescent protein
GSK: glycogen synthase kinase
HA: hemagglutinin
*myc*: myelocytomatosis oncogene
RACE: rapid amplification of cDNA ends
Tcf/LEF: T‐cell factor/lymphoid enhancer‐binding factor
Wnt: wingless/int

## Introduction

The c‐*myc* gene has been originally identified in the form of the transforming determinant of avian acute leukemia virus MC29 1, 2. The highly oncogenic v‐*myc* allele is derived from the cellular chicken c‐*myc* protooncogene by retroviral transduction 1, 2, 3, 4, 5. The cellular c‐*myc* proto‐oncogene encodes the c‐Myc protein, a transcription factor with oncogenic potential representing the central hub of a network controlling global gene expression and regulating fundamental cellular processes like growth, proliferation, differentiation, metabolism, and apoptosis 2, 3, 5, 6, 7, 8. c‐Myc is a bHLHZip protein encompassing protein dimerization domains (helix‐loop‐helix, leucine zipper) and a DNA contact surface (basic region) that forms heterodimers with the Max (MAX) protein and binds typically to specific DNA sequence elements termed E‐boxes (5′‐CACGTG‐3′) 4, 5. Upstream acting signaling pathways regulate the c‐Myc transcription factor network like those triggered by mitogenic receptor tyrosine kinases, or by wingless/int (Wnt) 9, 10, 11, 12. Human T‐cell factor 4 (Tcf4), the effector of Wnt/β‐Catenin signaling has been identified as an oncogenic regulator of the c‐*myc* and *cyclin D1* genes in colon cancer 13, 14, 15. The Wnt signaling pathway is highly conserved throughout animal evolution representing one of the key cascades to regulate development and stemness 14, 16, 17.

c‐Myc and Max homologs with conserved basic functions have been found in early diverging metazoans 18 and even in premetazoans 19, suggesting that principal functions of the c‐Myc master regulator arose very early in the evolution of multicellular animals. In the diploblastic cnidarian *Hydra*, two c‐*myc* homologs (*myc1* and *myc2*) have been identified, which are transcriptionally activated in the interstitial stem cell system 18, 20, 21. Both *Hydra* Myc proteins show the same degree of overall sequence identity (32%) compared to human c‐Myc, but Myc2 is structurally more related to its human ortholog concerning protein size and the degree of Myc box conservation (MBI, MBIII) in the transactivation domain 18. Paradoxically, downregulation of *myc1* by short interfering RNA or chemical inhibition promoted stem cell proliferation 22, suggesting a divergent role of Myc1 in the homeostasis of the interstitial stem cell lineage. In contrast to *myc1*, expression of the *myc2* gene is not restricted to the interstitial stem cell system but also occurs in proliferating epithelial stem cells throughout the gastric region. Furthermore, *myc2* is specifically activated in cycling precursor cells during early oogenesis and spermatogenesis, suggesting that the Myc2 protein has a possible nonredundant function in cell cycle progression 20.

Crucial components of the Wnt signaling pathway regulating c‐*myc* in vertebrates are conserved in *Hydra* such as Wnt3a, Frizzled, Dsh (disheveled), GSK (glycogen synthase kinase)‐3β, β‐Catenin, or Tcf (T‐cell factor)/Lef (lymphoid enhancer‐binding factor) 23, 24, 25, 26. Further studies have revealed a surprising complexity of cnidarian Wnt proteins that are implicated in fundamental morphogenetic processes emphasizing the important role of Wnt signaling in organismal patterning throughout the animal kingdom 16, 27, 28, 29, 30, 31. Here, we report that *myc1* is downregulated in the gastric region upon ectopic activation of β‐Catenin signaling, whereas under the same conditions *myc2* expression levels remain constant. Our results therefore imply that the *Hydra myc1* gene represents a potential negative target of the Wnt/β‐Catenin/Tcf signaling pathway and that *myc2* presumably represents the functional ortholog of human c‐*myc*.

## Results

### Activation of β‐Catenin signaling in *Hydra* leads to specific repression of *myc1*

**Figure 1:**
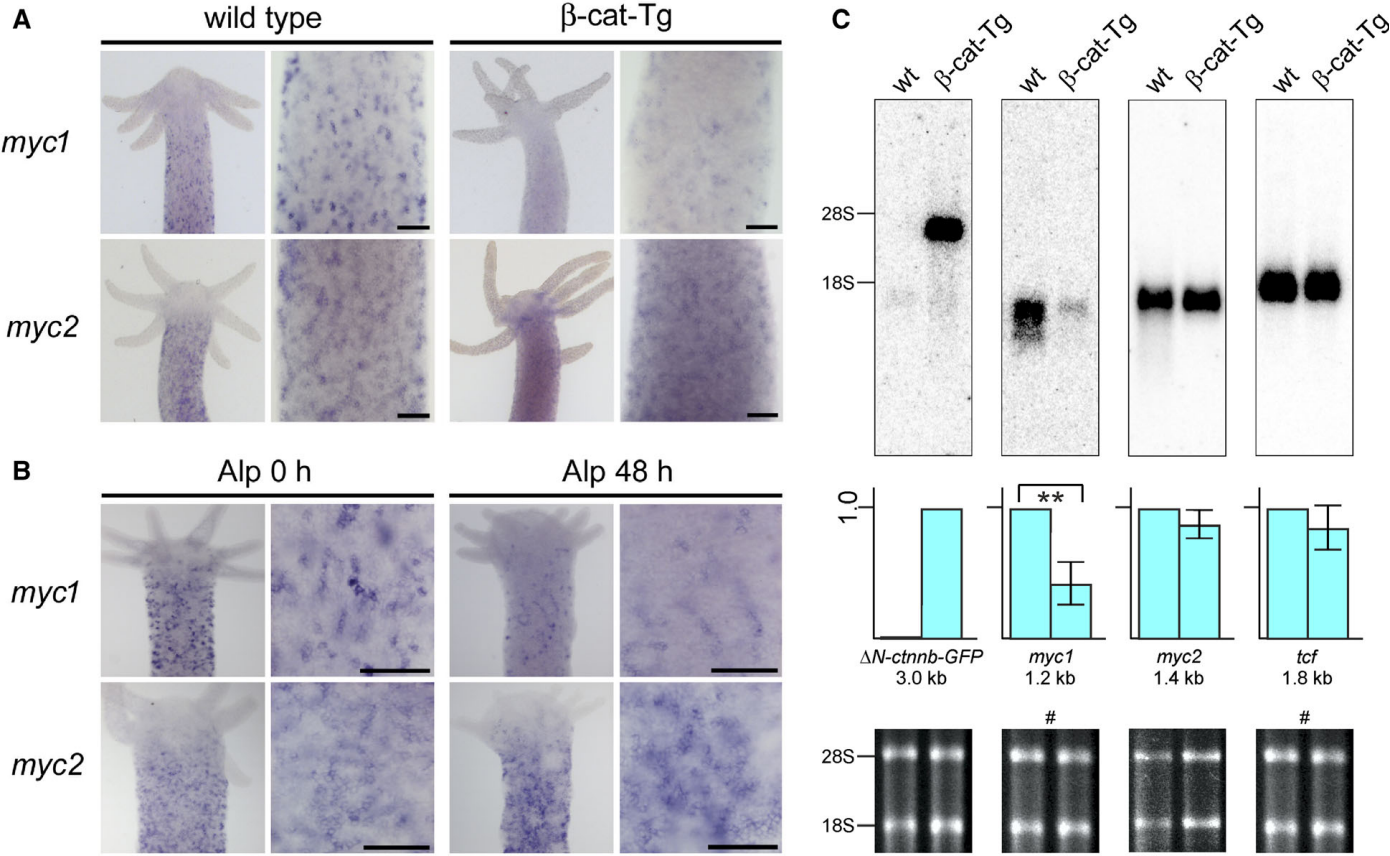
Specific repression of *myc1* mRNA expression upon β‐Catenin activation. (A) Expression patterns of *Hydra myc1* and *myc2* in β‐Catenin transgenic animals (β‐cat‐Tg) compared to wild‐type polyps. Whole wild‐type and transgenic polyps have *myc1* and *myc2* expressed throughout their body columns, but not in head and foot structures. *myc1* is downregulated in the transgenic polyps, whereas *myc2* levels are not significantly changed. (B) Differential expression of *Hydra myc1* and *myc2* 48 h after onset of treatment with the GSK‐3β inhibitor Alp. Inhibition of GSK‐3β results in activation of β‐Catenin in the canonical Wnt signaling pathway, and as a result in a reduction of the *myc1* expression level. Upper halves of polyps and magnified views from gastric regions are shown. Bars in the magnified views represent 25 μm. (C) Northern analysis using aliquots (2.0 μg) of poly(A)^+^‐selected RNAs from whole wild‐type (wt) and transgenic *Hydra* animals, and *Hydra myc1*,* myc2*, or *tcf*‐specific cDNA probes derived from the relevant coding regions. Positions of residual ribosomal RNAs (28S, 18S) are given on the left site. Ethidium bromide‐stained RNAs used for blot analysis are shown below. The blot, which was hybridized to a second probe after filter washing is indicated by a hash sign (#). For transgenic mRNA detection, a *GFP*‐specific probe (*ΔN‐ctnnb‐GFP*) was applied (arbitrary expression level 1.0). Representative blots from three independent experiments are shown where *myc1*,* myc2,* and *tcf* levels from wild‐type animals were arbitrarily set to 1.0. Standard deviations (SD,* n* = 3) are shown by vertical bars. Statistical significance was assessed by using a paired Student *t*‐test (**P < 0.01).

Possible effects of nuclear β‐Catenin signaling on the expression of the *myc1* and *myc2* genes were analyzed by *in situ* hybridization. Polyps from transgenic *Hydra* (β‐cat‐Tg) express high levels of a β‐Catenin‐GFP fusion protein triggered by an actin promoter throughout their entire body columns. As a result of the enhanced nuclear signaling activity of β‐Catenin in all cells, β‐cat‐Tg animals form multiple ectopic head and foot structures 31, 32. These animals were compared with wild‐type polyps by testing for *myc1* and *myc2* expression. The *in situ* hybridization patterns showed that *myc1* was significantly downregulated throughout the body column of β‐cat‐Tg animals, whereas *myc2* expression remained unchanged or even appeared to be slightly upregulated in distinct cells (Fig. 1A). Expression of *myc1* and *myc2* was also analyzed after Alsterpaullone (Alp) treatment, and compared with nontreated wild‐type control animals. This compound specifically blocks the activity of GSK‐3β that normally contributes to β‐Catenin degradation by phosphorylation 28. Consequently, β‐Catenin levels in cell nuclei are elevated due to protein stabilization, and ectopic tentacles and heads form along the entire body column 28, 30. Equivalent to the results obtained in β‐cat‐Tg animals, expression of *myc1* was downregulated after 48 h in Alp‐treated animals, whereas *myc2* expression showed the same expression pattern as observed for the β‐cat‐Tg animals (Fig. 1B).

**Figure 2:**
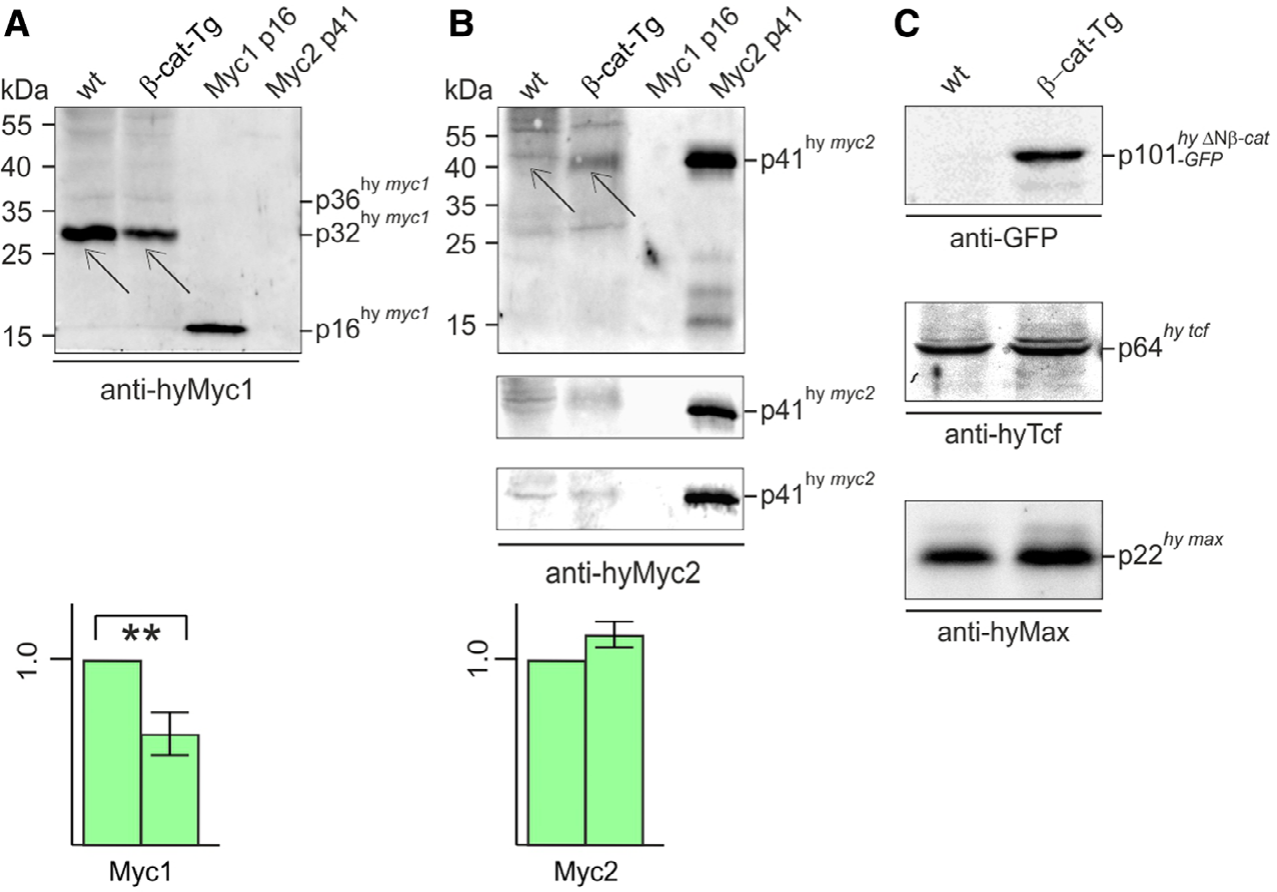
Specific repression of Myc1 protein expression upon β‐Catenin activation. (A, B) Expression of *Hydra* Myc1 (A) and Myc2 (B) proteins in β‐*catenin* transgenic animals (β‐cat‐Tg) compared to wild‐type (wt) polyps. (C) Expression control of the transgenic fusion protein ΔN‐Ctnnb‐GFP, and of *Hydra* Tcf or *Hydra* Max in β‐cat‐Tg compared to wt polyps. Protein expression was tested by immunoblot analysis using each 10 μg of cell extract. Proteins were resolved by SDS/PAGE (10%, wt/vol) and detected using antibodies directed against recombinant Myc1, Myc2, Max, or Tcf proteins, or against the green fluorescent protein (GFP). As specificity control, each 10 ng of recombinant Myc1 p16 or full‐length Myc2 (p41) were used in panels A and B. Representative blots from three independent experiments are shown, where Myc1 and Myc2 levels from wild‐type animals were arbitrarily set to 1.0. Thin arrows depict the endogenous *Hydra* Myc1 or Myc2 protein bands. Standard deviations (SD,* n* = 3) are shown by vertical bars. Statistical significance was assessed by using a paired Student *t*‐test (**P < 0.01). In case of Myc2, which is hardly detectable by immunoblotting, all three blots used for quantification are shown (*P* = 0.08).

To confirm the specific downregulation of *myc1*, poly(A)^+^‐selected RNAs isolated from whole β‐cat‐Tg animals and from whole wild‐type controls were analyzed by Northern hybridization using DNA probes derived from the coding regions of *Hydra myc1*,* myc2*, and *tcf*. As a control for detection of the transgenic *ΔN‐ctnnb‐GFP* mRNA, a probe derived from the GFP‐encoding portion was used. The Northern analysis confirmed that, compared to normal animals, the transgenic polyps contain distinctly lower amounts of *myc1* mRNA, in contrast to *myc2*, or *tcf* whose overall expression levels were almost not affected (Fig. 1C). To test *myc1* and *myc2* expression also at the protein levels, immunoblot analysis was performed using cell extracts from whole animals and immunoglobulin G‐purified antisera directed against Myc1, or Myc2. Previous immunoprecipitation analyses using extracts from ^35^S‐methionine pulse‐labeled *Hydra* and a Myc1‐specific antibody have detected the endogenous Myc1 protein with an apparent molecular mass of 36 kDa and a minor 32‐kDa protein, whereas *in vitro* translated full‐length Myc1 had a size of 39 kDa 18, 20. The 36‐kDa Myc1 protein presumably results from the usage of an alternative translation start site 18. On the other hand, endogenous Myc2 was expressed as a 41‐kDa protein having the same size as the *in vitro* translated full‐length product 20. Here, immunoblot analysis using equal amounts of nonlabeled extracts from wild‐type and transgenic *Hydra* detected Myc1 and Myc2 proteins with similar apparent molecular masses as the previously detected proteins from pulse‐labeled cells, but in case of Myc1 the 32‐kDa protein band was the dominant one and the 36‐kDa isoform only weakly expressed (Fig. 2). Myc2, expressed at low levels as reported previously 20, displayed an apparent molecular mass of 41 kDa as expected. The 32‐kDa Myc1 isoform, which was downregulated in transgenic polyps, could result from proteolytic processing of the 36‐kDa protein. Although the nature of the smaller Myc1 protein needs yet to be determined, the results are in line with those obtained by *in situ* and Northern expression analyses (Fig. 1). The lower amounts of endogenous Myc2 versus Myc1 are probably due to the higher instability of the Myc2 protein as reported previously 20. Titer and specificities of the applied polyclonal antibodies were controlled using the carboxyl‐terminal Myc1 p16 and the full‐length Myc2 p41 protein (Fig. 2A,B). As expected, expression of the ΔN‐ctnnb‐GFP protein was exclusively detected in transgenic *Hydra*, whereas Tcf or Max were expressed at almost equal levels both in wild‐type and in transgenic animals (Fig. 2C).

**Figure 3:**
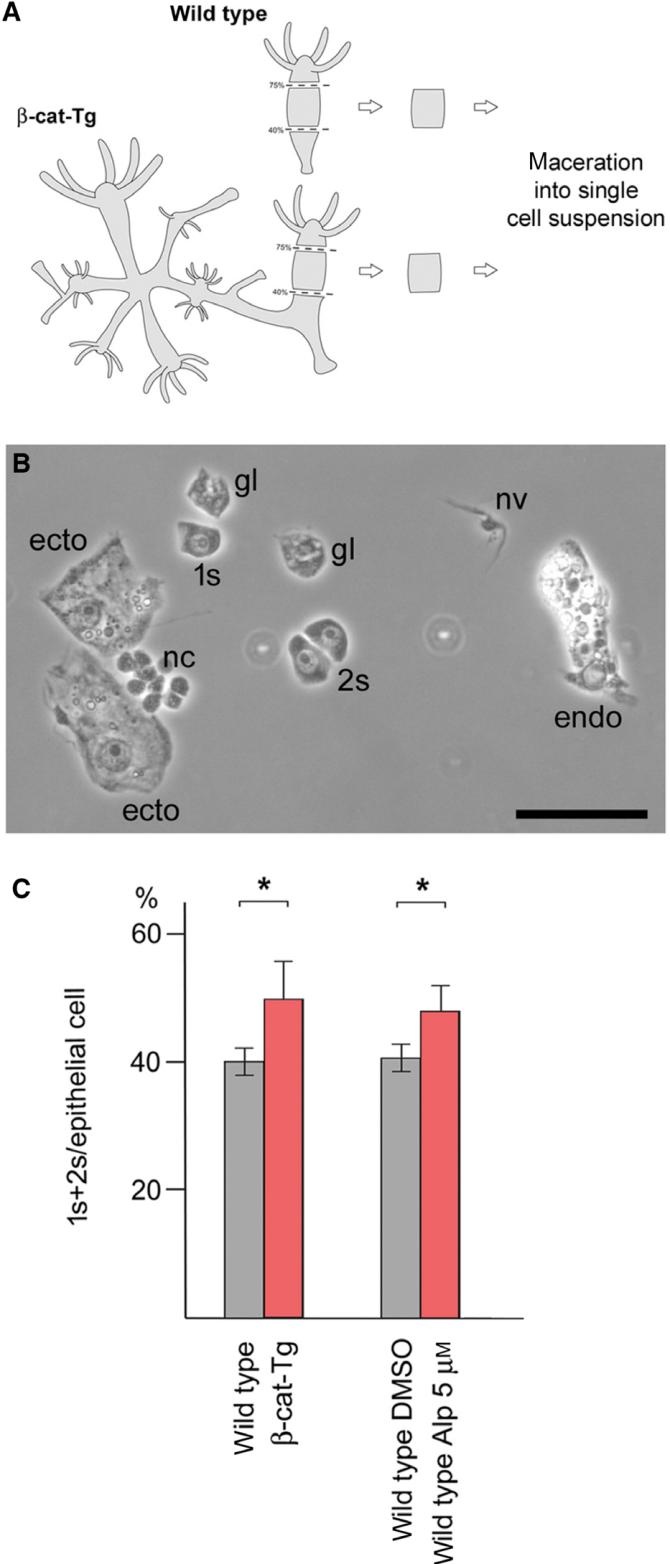
Interstitial stem cell densities in the body columns of β‐Catenin‐activated and wild‐type polyps. (A) Scheme of the position of macerated tissue pieces. Tissue samples representing roughly half of the tissue mass of a budless control polyp were excised from the mid body column. Equivalent tissue pieces were excised from the body columns of β‐cat‐Tg transgenes and polyps treated with Alp for 60 h. The tissue pieces were macerated, spread as single‐cell suspensions onto microscope slides, and analyzed using a phase contrast microscope. (B) Representative phase contrast image of macerated cells from a β‐cat‐Tg polyp. 1s: single interstitial stem cell; 2s: interstitial stem cell pair; ecto: ectodermal epithelial cell; endo: endodermal epithelial cell; nv: nerve cell; nc: nest of differentiating nematocytes; gl: gland cell. Bar: 25 μm. (C) Quantification of macerated tissue pieces reveals higher interstitial stem cell (1s+2s) densities in β‐Catenin‐activated tissues as compared with wild‐type controls. 1s+2s density is defined as the numbers of 1s+2s per epithelial cells. Bars represent the mean ± SD of three independent experiments. Statistical significance was assessed by using a paired Student *t*‐test (*P < 0.1).

Loss of *myc1* expression could result from either transcriptional downregulation of this gene in interstitial cells including stem cells, which occur as single cells (1s) and cell pairs (2s), or from potential β‐Catenin‐induced disappearance of *myc1*‐expressing cells. We studied putative changes in the numbers of *myc*‐expressing interstitial stem cells in the body columns of β‐Catenin‐activated versus wild‐type polyps using the maceration technique (Fig. 3A). In a macerated cell suspension, cells maintain their original morphology, and thereby the different cell types can be clearly distinguished and precisely quantified (Fig. 3B) 33. The results in Fig. 3C show that in both, β‐cat‐Tg and Alp‐treated tissue, interstitial stem cells are embedded at higher densities in the ecto‐ and endodermal epithelial tissue layers. Thus, enhanced nuclear β‐Catenin signaling seems to stimulate interstitial stem cell maintenance. Furthermore, our results clearly support the view that loss of *myc1* expression is most likely caused by β‐Catenin‐regulated transcriptional repression in these cells.

### *Hydra* Tcf binds to the promoters of *Hydra myc1* and *myc2*

**Figure 4:**
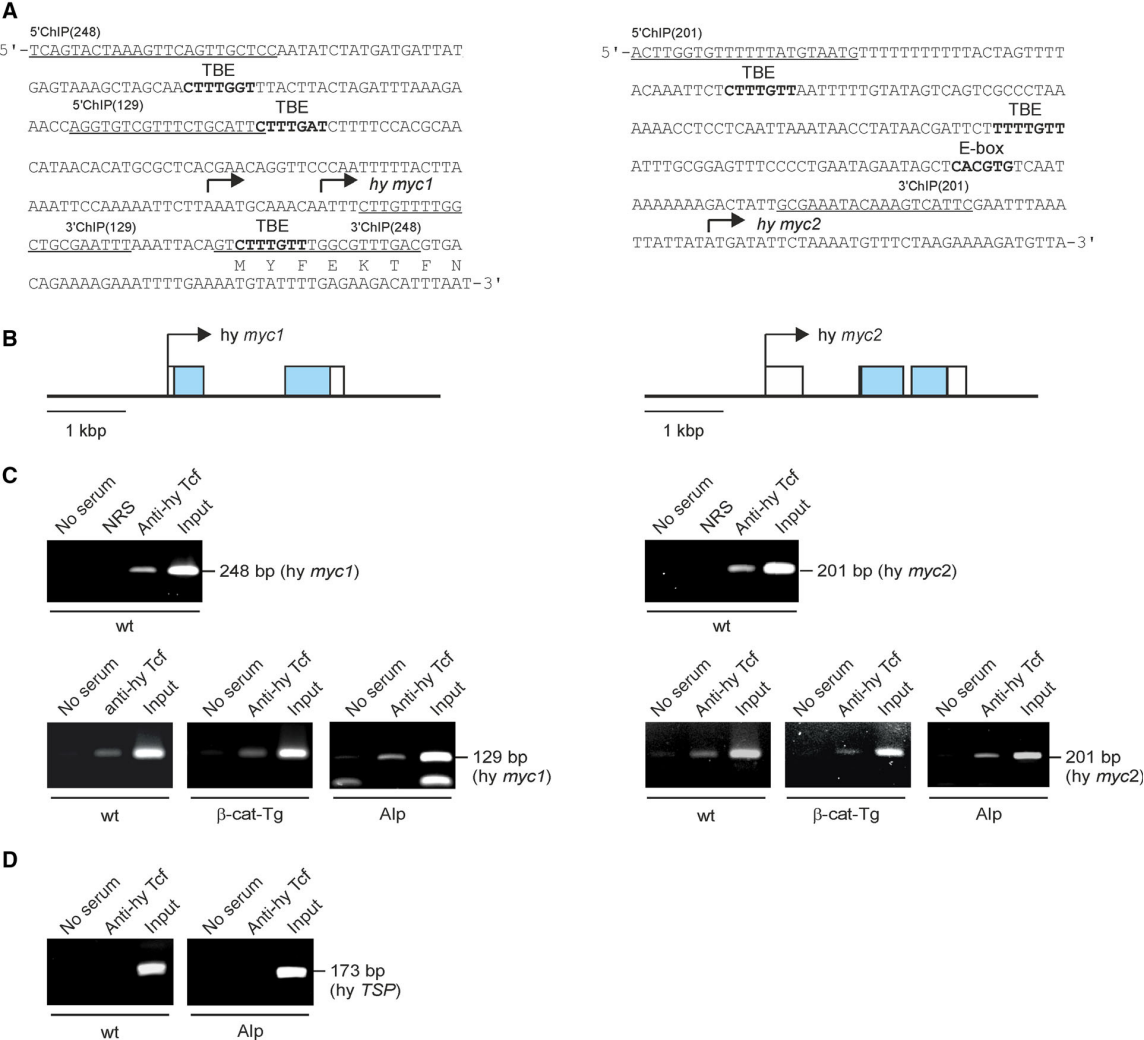
Structures of the *Hydra myc1* and *myc2* promoter regions and binding of *Hydra* Tcf. (A) Nucleotide sequences of the *myc1* and *myc2* regulatory regions. The transcription start sites mapped by 5′RACE (arrows), potential binding sites (in bold) for the transcription factors Tcf (TBE) or Myc (E‐box), and binding sites for 5′ and 3′ ChIP primers (underlined) are indicated. (B) Topographies of *Hydra* *magnipapillata* genomic loci (accession nos. NW_004167287, NW_004167363) containing the *Hydra myc1* and *myc2* genes. Exons are depicted by boxes with the coding regions shown in black. Arrows indicate the main transcription start sites. (C) ChIP of the *Hydra myc1* and *myc2* promoter regions using chromatin from whole *Hydra* wild type (wt) animals, from β‐*catenin* transgenic animals (β‐cat‐Tg), and from Alsterpaullone‐treated (Alp) *Hydra*. An antiserum directed against *Hydra* Tcf was used for precipitation, followed by PCR amplification of the indicated fragments from the *myc1* or *myc2* regulatory regions. Reactions with normal rabbit serum (NRS) or total chromatin (Input) were used as controls. (D) A fragment containing no Tcf binding site derived from the *Hydra TSP* regulatory region 36 was amplified as a control.

**Figure 5:**
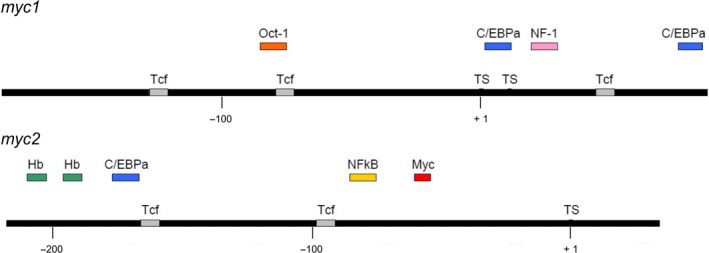
Promoter maps of *Hydra myc1* and *myc2*. Bars represent the promoter regions. Positions of transcription factor binding sites were identified using the computer program AliBaba2 (gene‐regulation.com) and are depicted on or above the bars (Tcf, T‐cell‐specific transcription factor; C/EBPalpha, CCAAT enhancer‐binding protein alpha; NF‐1, nuclear factor 1; Hb, hunchback; NFkappaB, nuclear factor kappa B; Myc, myelocytomatosis viral oncogene protein product).

To investigate the mechanism of *Hydra myc1* downregulation by Wnt/β‐Catenin signaling, the promoter region of *myc1* was defined by transcription start site mapping. cDNA prepared from whole *Hydra* was subjected to 5′rapid amplification of cDNA ends (5′RACE). Two closely spaced transcription start sites were detected in the *myc1* promoter region, whereas transcription of *myc2* starts at one defined site (Fig. 4A) as reported recently 20. The sequence of the *myc1* 5′RACE product also allowed deduction of the relevant full‐length mRNA sequence. Alignment of this sequence with the published *Hydra* genome 34 led to definition of the *myc1* gene topography. In contrast to *myc2*, which consists of three exons similar to the vertebrate c‐*myc* genes 20, *Hydra myc1* has two exons only (Fig. 4B). Inspection of the 5′‐untranscribed regions of *myc1* and *myc2* revealed that both promoters contain potential binding elements termed TBE 13 for the Tcf transcription factor, closely matching the consensus site 5′‐CCTTTGWW‐3′ 35 (Fig. 4A). Whereas the *myc2* promoter contains two TBE motifs located at positions −166 and −98 nucleotides (nt) upstream of the transcription start site, three canonical TBE sites are present in the *myc1* promoter mapping to nt positions −128, −79, and +46 with reference to the proximal transcription start site (Fig. 4A). Interestingly, the *myc2* promoter also contains a canonical Myc binding site (E‐box) at nt position −60 (Fig. 4A). Inspection of the promoters for further transcription factor binding sites revealed the presence of Oct‐1, C/EBPα, or NF‐1 motifs in the *myc1*, and of *hunchback*, C/EBPα, or NFκB motifs in the *myc2* promoter, respectively (Fig. 5).

**Figure 6:**
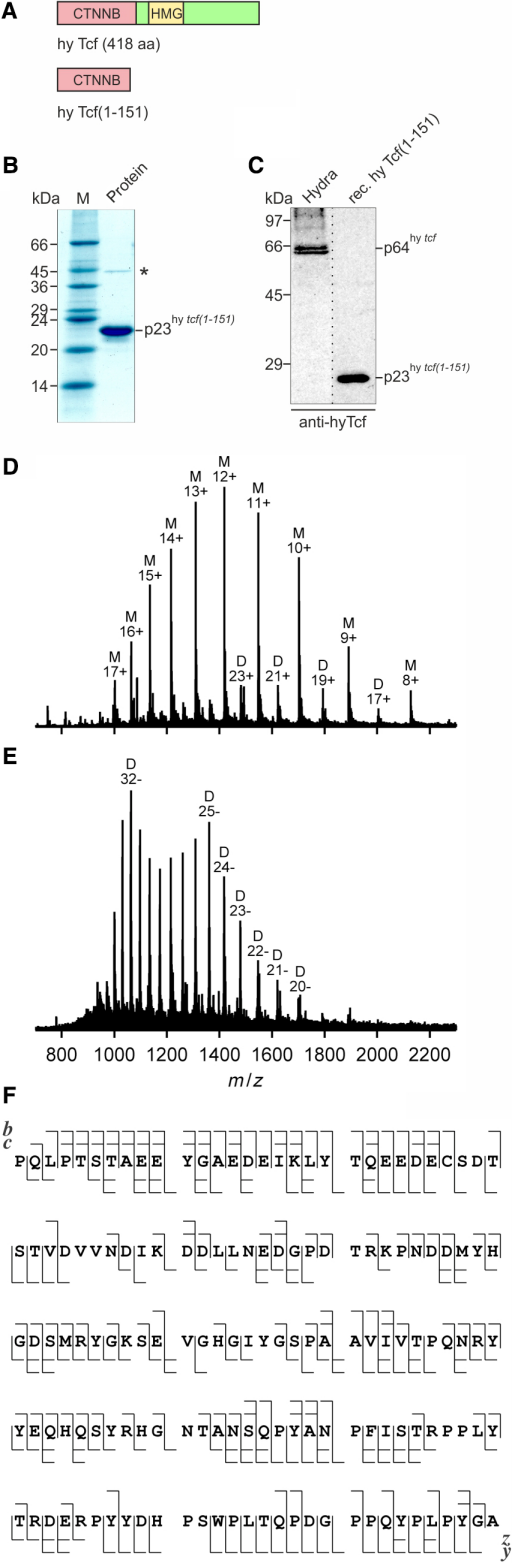
Analysis of the *Hydra* Tcf recombinant protein. (A) Schematic depiction of the *Hydra* (hy) Tcf protein product (GenBank accession no. XP_002159974). The positions of the high mobility group (HMG) representing the DNA binding domain, and the β‐Catenin binding site (CTNNB) are indicated. The amino‐terminal segment of *Hydra* Tcf [hy Tcf(1‐151)] was expressed in *Escherichia coli* and purified. (B) SDS/PAGE (12.5%, wt/vol) of 5 μg (Coomassie brilliant blue staining) purified recombinant *Hydra* Tcf(1‐151) p23. The upper faint band marked with an asterisk represents a cross‐linked dimer generated from p23^hy *tcf(1‐151)*^ (see below). (C) Immunoblot analysis using 10 μg of cell extract from *Hydra*, and 5 ng of recombinant hy Tcf(1‐151). Proteins were resolved by SDS/PAGE (10%, wt/vol) and detected using an antiserum directed against recombinant hy Tcf(1‐151). The dotted line marks the splicing site in the blot image, from which one lane has been removed. (D–F) MS of recombinant hy Tcf(1‐151). The ESI‐MS of hy Tcf(1‐151) with a 7 Tesla Fourier transform ion cyclotron resonance (FT‐ICR) instrument (Bruker, Vienna, Austria) gives a mass value for the most abundant isotopic peak of 17 014.868 ± 0.006 Da (theoretical mass without initiating methionine: 17 014.874 Da; error 0.4 p.p.m.) using polyethylene glycol 1000 as calibrant. (D) ESI mass spectrum of hy Tcf‐NT in positive ion mode showing mostly (~ 95%) monomeric protein (M) but also protein dimers whose mass values (measured mass for the most abundant isotopic peak 34 027.727 Da) indicate covalent dimerization by formation of an intermolecular disulfide bond (theoretical mass for the most abundant isotopic peak 34 027.732 Da). (E) ESI of hy Tcf‐NT in negative ion mode gives predominantly (> 80%) dimer ions from intermolecular disulfide bond formation. (F) Fragment ion maps for the *Hydra* Tcf(1‐151) p23 protein showing 85% sequence coverage.

To test if promoter regions of *Hydra myc1* and *myc2* are bound by Tcf *in vivo*, ChIP analysis was performed using cross‐linked chromatin from whole wild‐type, transgenic, and Alp‐treated *Hydra* animals, and a Tcf‐specific antibody followed by PCR amplification of the specific DNA regions (Fig. 4C). To generate the antiserum directed against *Hydra* Tcf, a partial recombinant *Hydra* Tcf protein was expressed in *Escherichia coli*, purified, and then used as an immunogen (Fig. 6). Bound *Hydra* Tcf was detectable on both promoter segments (Fig. 4C) suggesting that a β‐Catenin/Tcf protein complex could indeed regulate the *myc1* and *myc2* genes. No significant changes in promoter occupancy were observed after β‐Catenin activation either by overexpression (β‐cat‐Tg) or GSK‐3β inhibition (Alp) (Fig. 4C). As a negative control for Tcf binding, a segment derived from the *Hydra* thrombospondin (*TSP*) promoter containing no Tcf binding site 36 was tested (Fig. 4D).

### The *myc1* promoter is selectively downregulated by ectopic β‐Catenin/Tcf

**Figure 7:**
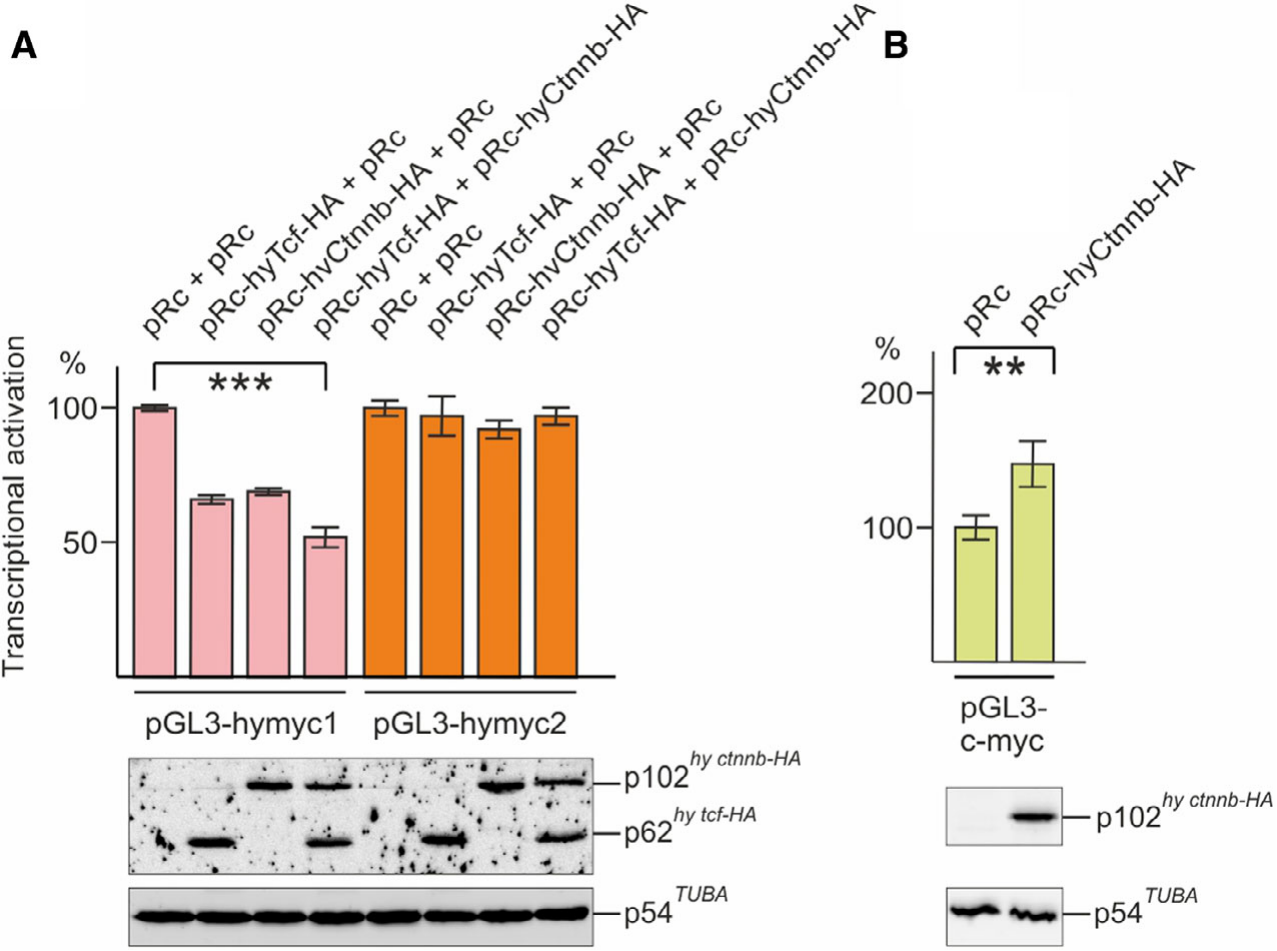
Transcriptional regulation of the *Hydra myc1* and *myc2* promoters. (A) Aliquots (0.5 μg) of the pGL3‐hymyc1 or pGL3‐hymyc2 reporter constructs were co‐transfected in triplicate with aliquots (0.5 μg) of pRc‐derived expression vectors encoding the *Hydra* Tcf‐HA, Ctnnb‐HA, or the empty expression vector (pRc) into the chemically transformed quail cell line QT6. (B) Aliquots (1.0 μg) of the pGL3‐c‐myc reporter construct were co‐transfected in triplicate with aliquots (1.0 μg) of pRc‐hyCtnnb‐HA, or the empty pRc vector into QT6 cells. Luciferase activities and protein concentrations were determined from cell extracts prepared 24 h after transfection. Relative luciferase activities and standard errors of the mean (SEM,* n* = 3) are visualized by bars and vertical lines, respectively. Statistical significance was assessed by using a paired Student *t*‐test (**P < 0.01, ***P < 0.001). For control of protein expression (lower panels), equal amounts of cell extracts (20 μL) were analyzed by SDS/PAGE (10% wt/vol). The ectopic HA‐tagged *Hydra* Tcf and Ctnnb proteins, and endogenous tubulin α were detected by immunoblotting.

To test if the *Hydra* β‐Catenin and Tcf proteins are involved in transcriptional regulation of the *myc1* and *myc2* genes, luciferase reporter assays were performed using the chemically transformed quail cell line QT6 as test system. Cells were transiently co‐transfected with reporter plasmids containing the *Hydra myc1* and *myc2* promoters inserted into luciferase reporter vectors (pGL3‐hymyc1, pGL3‐hymyc2), and expression vectors (pRc) encoding *Hydra* Tcf or β‐Catenin proteins fused to a carboxyl‐terminal hemagglutinin (HA) tag (Fig. 7A). Ectopic protein expression was verified by immunoblotting. Overexpression of Tcf‐HA, β‐Catenin‐HA, or Tcf‐HA together with β‐Catenin‐HA led to partial repression of the *myc1* promoter, whereas the *myc2* promoter was not influenced. The results suggest that, in contrast to *myc2*, the *myc1* promoter is repressed by β‐Catenin/Tcf although both promoters are bound by Tcf *in vivo* (Fig. 4C). Therefore, these biochemical analyses confirm the specific downregulation of *myc1* upon activation of the β‐Catenin/Tcf signaling complex suggesting that *myc2* but not *myc1* represents the functional ortholog of vertebrate *c‐myc*.

**Figure 8:**
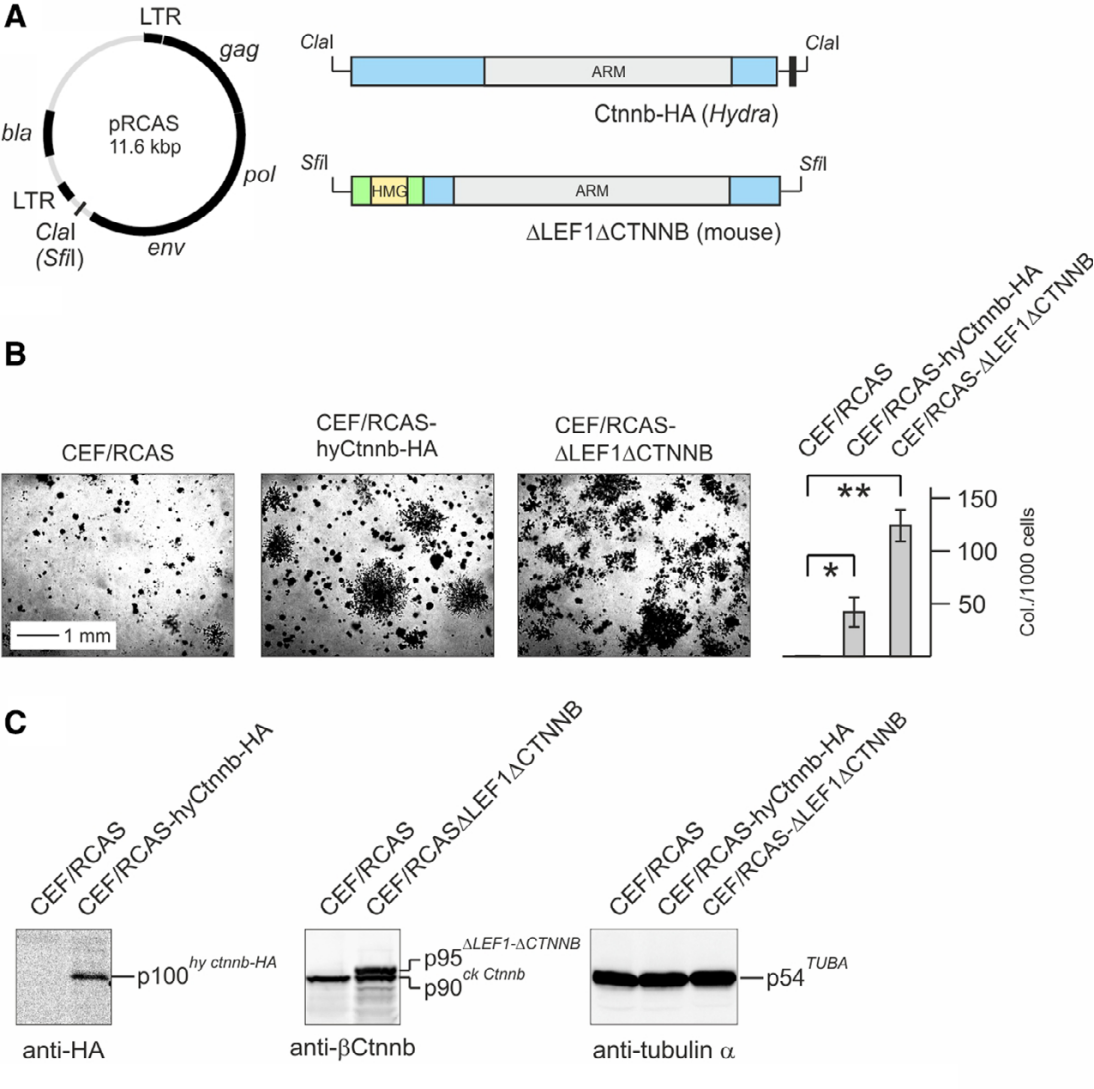
Oncogenic transforming activity of *Hydra* β‐Catenin. (A) Structures of the applied RCAS constructs (HA, hemagglutinin tag). (B) Agar colony formation of CEF transfected with the empty retroviral RCAS vector, or with RCAS constructs depicted under A. Each 1 × 10^4^ cells were seeded in soft nutrient agar onto MP12 dishes and colonies were scored after 3 weeks. Untransformed control CEF infected with the empty RCAS vector produced very small background colonies, which has been also observed previously 37, 38. Colonies were counted from triplicate dishes. A representative experiment from two independent assays (*n* = 2) is shown. Vertical bars show standard deviations (SD). Statistical significance was assessed by using a paired Student *t*‐test (*P < 0.05, **P < 0.01). (C) Immunoblot analysis of ectopically expressed proteins using the indicated antibodies. The antibody directed against the mouse β‐Catenin portion present in the ΔLEF1ΔCTNNB fusion protein also detects the endogenous chicken (ck) Ctnnb (p90).

To test, if *Hydra* β‐Catenin is capable at all to activate transcription in the applied cell system, a reporter construct containing the chicken c‐*myc* promoter containing multiple Tcf binding sites was co‐transfected with pRc‐hyCtnnb‐HA into QT6 cells. The result of this luciferase assay showed that, in contrast to the *Hydra myc1* or *myc2* promoters, *Hydra* β‐Catenin moderately activates this vertebrate c‐*myc* promoter (Fig. 7B). No luciferase activities were scored upon transfection of either the empty reporter plasmid, or the empty expression vector (data not shown). To investigate if overexpressed *Hydra* β‐Catenin also induces cell transformation in avian cells similar to its vertebrate counterparts 37, 38, the coding region of *Hydra* β‐*catenin* was inserted into the replication‐competent retroviral RCAS expression vector (Fig. 8A) and transfected into chicken embryo fibroblasts (CEF). As a positive control, an RCAS construct encoding a LEF1/β‐Catenin fusion protein (ΔLEF1ΔCTNNB) 37, 38 was employed. As expected, overexpression of ΔLEF1ΔCTNNB lead to efficient cell transformation manifested by colony formation in soft agar 37, 38 (Fig. 8B). Remarkably, also the cells overexpressing full‐length *Hydra* β‐Catenin were able to form colonies indicating a transformed phenotype (Fig. 8B). The ectopic proteins were efficiently expressed from their retroviral vectors, which was monitored by immunoblot analysis (Fig. 8C). This result shows that the principal oncogenic potential of β‐Catenin has been conserved through metazoan evolution.

## Discussion

Although substantial progress has been made in understanding the pleiotropic functions of the human c‐Myc protein in cellular proliferation, growth, energy metabolism, differentiation, and apoptosis 5, 6, 12, 39, many open questions remain regarding the underlying molecular mechanisms. The recent identification of c‐Myc to act as a possible general amplifier of gene expression controlling multiple transcriptional programs 8, 40, 41, 42, 43 even enhances the complexity of Myc biology. Furthermore, several upstream signaling pathways like the mitogenic Ras/Raf cascade, or the Wnt/β‐Catenin/Tcf4 axis regulate c‐Myc expression and activity in cell proliferation, but they are also relevant in malignant cell growth leading to aberrant c‐*myc* activation 2, 4, 5, 12.

A possibility to dissect the multiple Myc functions is the analysis of genetically defined invertebrate model organisms, like the simple eumetazoan *Hydra*, a classical diploblastic model system to study pattern formation, regeneration, and stem cell dynamics. About 600 million years ago, single‐cell premetazoans and simple metazoans were predominant, but during the Cambrian period, a rapid diversification of multicellular lifeforms began. *Hydra* represents one of the earliest multicellular animals to evolve out of single‐celled basal species, has a high regeneration potential, and is biologically immortal 21. This ancient organism could represent a suitable model system to study basal mechanisms in tumor biology because cancer is as old as multicellular life. Intriguingly, natural occurring tumors have been observed in *Hydra*, presumably resulting from differentiation‐arrested female gametes 44, a cell type in which *myc2* is expressed at high levels 20. The occurrence of tumors in this early diverging organism is actually in line with the atavistic cancer model, in which the biological origin of malignant growth is traced back to the transition between unicellularity to multicellularity 45, 46. This theory was recently supported by molecular analyses of multiple sets of genes with common phylogenetic origin (phylostrata) in solid human tumors. The study showed that dormant genes conserved with unicellular organisms became strongly upregulated in tumors, whereas genes of metazoan origin were primarily inactivated 47.

*Hydra* is also one of the most basal metazoan organisms employed so far for analysis of the major cancer driver Myc and its signaling network 18, 20 revealing that biochemical and oncogenic properties of c‐Myc arose very early in metazoan evolution. In *Hydra* there are four *myc*‐related genes (*myc1–4*), which branched off from a basal position during cnidarian evolution 18, 20, 21. *myc1* and *myc2* show the closest homology to vertebrate c‐*myc* but significantly less homology to L‐*myc* or N‐*myc* 18, because the diversification of c‐, L‐, and N‐*myc* subfamilies occurred later within the vertebrate lineage 18. On the protein level, *Hydra* Myc1 shares 32%, 22%, or 20% amino acid sequence identity with the human c‐Myc, L‐Myc, or N‐Myc proteins, whereas Myc2 displays 32%, 22%, or 24% identity with c‐Myc, L‐Myc, or N‐Myc, respectively. As reported previously, the structures of *Hydra* Myc1 and Myc2 proteins display the same principal topography and similar evolutionary relationship with the human c‐Myc protein with equal sequence identities (32%), but share only 24% overall identities among themselves 18. Although Myc1 or Myc2 cannot be assigned directly as ancestor of any specific vertebrate homolog, comparison of the sizes and conserved Myc boxes in the transactivation domain between *Hydra* Myc1 or Myc2 and human c‐Myc suggests that Myc2 is most likely the closest ortholog of human c‐Myc 18. Furthermore, the *myc2* gene is composed of three exons like human c‐*myc*, in contrast to *myc1* containing two exons only (Fig. 4B). Concerning the differential regulation of the *Hydra myc* genes (Fig. 7), comparison of the promoter structures indicates, that besides Tcf/β‐Catenin additional transcription factors may be involved in mediating transmission of upstream acting signaling pathways (Fig. 5). In addition, the presence of a Myc binding site in the *myc2* promoter suggests that *Hydra* Myc proteins themselves may participate in *myc2*‐specific gene regulation.

**Figure 9:**
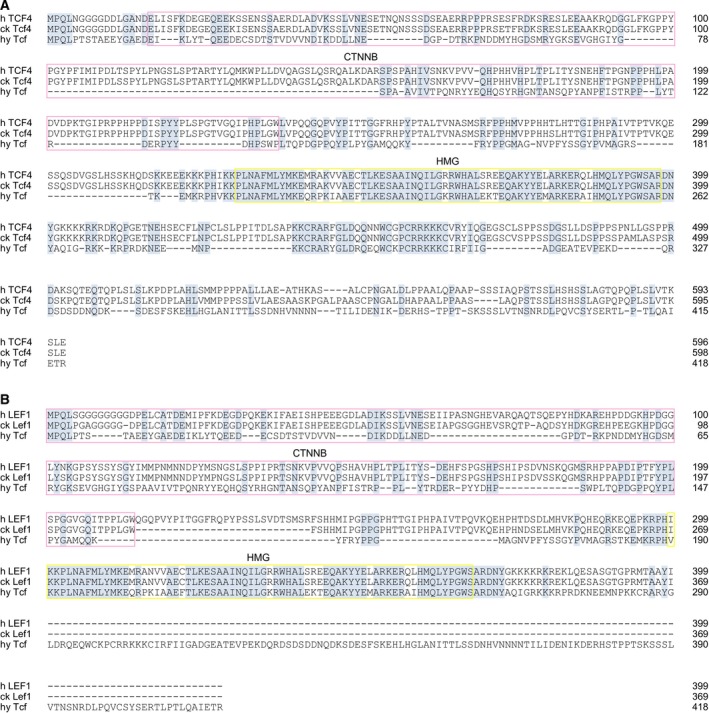
Amino acid sequence alignments of *Hydra magnipillata* (hy) Tcf with the *Homo sapiens* (h) and *Gallus gallus* (ck) homologs. (A) Alignment with human TCF4 (TCF7L2) and chicken Tcf4 (Tcf7l2). (B) Alignment with human LEF‐1 and chicken Lef1. GenBank accession numbers are: hy Tcf, NP_001296662; h TCF‐4, CAG38811; ck Tcf‐4, NP_001193439; h LEF‐1, Q9UJU2; ck Lef‐1, NP990344. Identical residues are shaded in blue, and gaps are indicated by dashes. The β‐Catenin (CTNNB) and DNA binding (HMG) domains are boxed in pink or yellow, respectively. Sequence identities between hy Tcf and h LEF1 or ck Lef1 are 29%, and between hy Tcf and h TCF4 or ck Tcf4 42%. The alignment was generated by using the computer program (omega) clustalw with additional manual adjustments.

**Figure 10:**
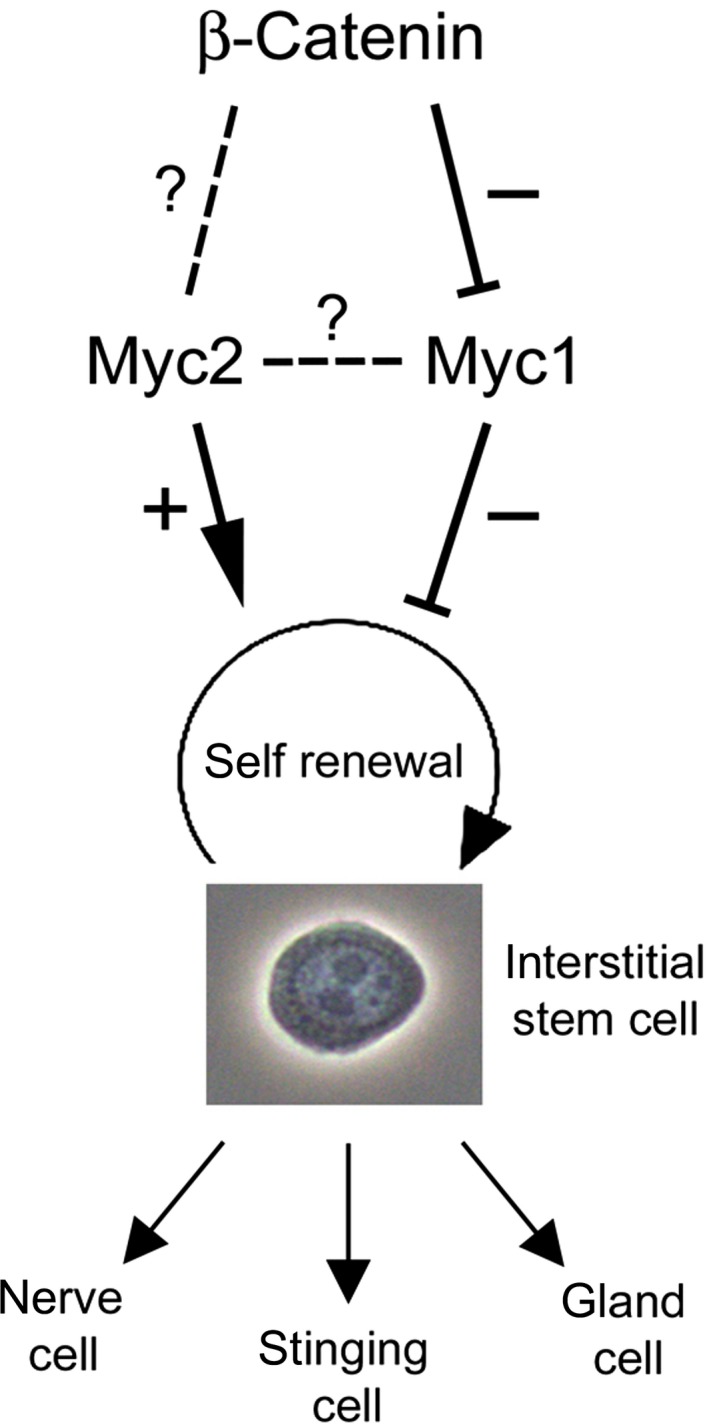
*Hydra* β‐Catenin/Myc interaction model. Interstitial stem cells permanently undergo self‐renewal and differentiation into three somatic products: nerve cells, stinging cells (nematocytes), and gland cells. We propose that Myc2 acts as maintenance factor in interstitial stem cell self‐renewal and that this is complemented by a double‐negative action of β‐Catenin and Myc1. The detailed actions of β‐Catenin on Myc2 and the cross‐talk between Myc1 and Myc2 are not yet known.

In mammalian Wnt/β‐Catenin signal transduction, the transcription factor Tcf4 represents a crucial nuclear effector 9, 14. Comparison of the *Hydra* Tcf protein sequence with homologs from chicken or human reveals the highest degree of sequence identity with the vertebrate Tcf4 (Fig. 9), regulating vertebrate c‐*myc* via TBE elements present in the relevant promoter regions 13. In *Hydra*,* wnt* and *tcf* genes are transcriptionally activated in early bud formation and head regeneration namely in the putative *Hydra* head organizer, the upper part of the hypostome 23, 28. Furthermore, elevated levels of β‐Catenin result in body columns that have high head organizer potency accounting for the formation of ectopic head structures in these animals 28 (Fig. 1A,B). Here, we present evidence that β‐Catenin has an additional function in interstitial cells of the gastric region. Using pharmacologically treated or genetically modified *Hydra*, we have shown that activation of β‐Catenin seems to enhance interstitial stem cell maintenance, and expression and promoter analyses demonstrate a specific repression of *myc1,* but not of *myc2*. According to our current working model (Fig. 10), this reduction in *myc1* expression by nuclear β‐Catenin could in fact promote interstitial stem cell maintenance and self‐renewal based on an earlier observation that *myc1* inhibition by *myc1*‐specific antisense RNAs has the same effect 22. The reason why in our biochemical analyses *myc2* is not significantly induced upon ectopic β‐Catenin/Tcf activation is not yet clear. So far, our experiments were performed in intact polyps only. Therefore, it was not possible to resolve how *myc2* expression responds to changes in nuclear β‐Catenin activity specifically in interstitial cells. A reason for the overall unchanged *myc2* expression could be that *myc2* is indeed activated in stem cells, but this is masked by a possible downregulation in epithelial cells. Since the two epithelial cell types form the major part of the *Hydra* body mass, a slight reduction in *myc2* expression upon β‐Catenin activation could dilute a positive stimulatory effect in interstitial stem cells. Subtle shifts in Myc expression above or below distinct thresholds can trigger striking differences in biological output 48. In addition, *myc2* expression may depend on possible Myc1‐triggered transcriptional activation mediated by the E‐box present in the *myc2* promoter (Fig. 4A). Consequently, a decrease in Myc1 levels due to Wnt signal‐triggered *myc1* downregulation could mask a possible stimulatory effect of β‐Catenin/Tcf on the *myc2* promoter, a hypothesis, which has to be pursued by further investigations.

Our finding that *Hydra myc1* is a negatively regulated target of β‐Catenin was unexpected, because c‐*myc* is one of the best studied targets activated by mammalian β‐Catenin. However, also in case of human N‐*myc*, a negative regulation of mRNA expression caused by activated Wnt signaling has been reported 49, 50. In murine embryonic stem cells, c‐Myc controls the balance between stem cell self‐renewal, pluripotency and differentiation 51, 52 thereby driving a regulatory network to maintain this stem cell identity 53. Furthermore, c‐Myc potentiates the Wnt/β‐Catenin signaling pathway by transcriptional repression of Wnt antagonists. This induces transcriptional activation of the endogenous *myc* family members, which in turn activate a Myc‐driven self‐reinforcing circuit 54. Depletion of c‐*myc* by gene knock‐out or by inactivation of a c‐*myc*‐driving superenhancer induces reversible dormancy in stem cells with differentiation‐arrested pluripotent progenitors 55, 56.

In colon cancer, mutation of the APC tumor suppressor activates β‐Catenin/Tcf signaling, leading to transcriptional induction of c‐*myc* and *cyclin D1* in colon cancer 13, 15 illustrating the oncogenic potential of this signal transduction pathway. The ancestral *Hydra* β‐Catenin protein is highly conserved sharing 62% sequence identity to its human ortholog 23. Furthermore, *Hydra* β‐Catenin displays oncogenic potential similar to its vertebrate counterparts (Fig. 8). As reported previously, both *Hydra* Myc proteins have transforming activities 18, 20 demonstrating that structural elements with basic oncogenic properties must have been established very early in metazoan evolution. Further investigations involving appropriate transgenic and interfering RNA technologies will be performed to functionally characterize these transforming proteins in *Hydra*, and to fully elucidate upstream and downstream acting Myc signaling pathways.

## Materials and methods

### Animals

*Hydra vulgaris* strains Basel, *Hydra magnipapillata* wild‐type strain 105 and the *Hydra* β‐cat‐Tg strain containing a transgenic β‐*catenin* gene fused in frame to the coding sequence of the enhanced GFP 31 were used in this study. Mass cultures were kept as described 57. Experimental animals were collected 24 h after the last feeding. Treatment with the inhibitor Alp was done as described 28, and phenotypes of treated polyps were analyzed at 48 or 60 h after the onset of treatment.

### Maceration of *Hydra* tissue

Maceration of excised tissue pieces from the gastric region was done as described using a maceration solution containing acetic acid (1 vol)/glycerol (1 vol)/water (7 vol) 33. Fixed macerated cell suspensions were then spread onto microscope slides, and analyzed under phase contrast optics. For a single measurement, 10 tissue pieces were macerated, and about 500 epithelial cells and a corresponding number of 1s+2s interstitial stem cells were counted.

### Whole mount *in situ* hybridization

*In situ* hybridization with digoxigenin‐labeled RNA probes was done according to a protocol as described 58 using *myc1*‐ and *myc2*‐specific cDNA probes 18, 20.

### DNA cloning and nucleic acid analysis

Molecular cloning, DNA sequencing, and Northern analysis have been described 18, 59. Total RNA isolation and poly(A)^+^‐RNA selection from ~ 600 *Hydra* animals was performed as described 18. The yield of total RNA per animal was ~ 2 μg. DNA fragments specific for *Hydra myc1*, and *myc2* have been described 18, 20. To detect the *Hydra tcf* mRNA, a DNA fragment encompassing the *tcf* coding regions was applied. To obtain a DNA probe for detection of the transgenic *ΔN‐ctnnb‐GFP* mRNA, a 718‐nt *Eco*RI/*Sma*I fragment containing almost the entire GFP coding region was excised from the HoTG plasmid 60. To determine the *myc1* transcription start site, 5′RACE was performed as described 61 using the primers 5′‐GGATCATCATTAGTTGGAAATGGCGAAG‐3′ and 5′‐GTCTCGCAGTACTTCTGAAGGAAAAACACTTG‐3′ for first strand cDNA synthesis and subsequent PCR, respectively. Mapping of the *myc2* transcription start site using the primers 5′‐GTGTACACCAATTTGAACCAGTCATATCGA‐3′ and 5′‐AATTTATCCACAGCTATTATGTACACAATT‐3′ has been described previously 20.

### Promoter analysis

ChIP analysis was carried out as described 20, 59, 61, 62 using sheared extracts from ~ 300 *Hydra* animals treated with formaldehyde for 30 min. Immunoprecipitations were performed with specific antibodies followed by PCR amplification of 248‐ and 201‐bp fragments from the *Hydra myc1* or *myc2* regulatory regions, respectively, using the specific primer pairs 5′‐TCAGTACTAAAGTTCAGTTGCTCC‐3′/5′‐GTCTTTGTTTGGCGTTTGAC‐3′ (*myc1*), and 5′‐ACTTGGTGTTTTTTATGTAATG‐3′/5′‐GAATGACTTTGTATTTCGC‐3′ (*myc2*). In addition, the primer pair 5′‐AGGTGTCGTTTCTGCATTC‐3′/5′‐AAATTCGCAGCCAAAACAAG‐3′ (*myc1*) was employed to amplify a 129‐bp fragment from the *myc1* promoter region. The primers used to amplify a 173‐bp segment from the *TSP* promoter have been described 36.

Transcriptional transactivation analysis using the luciferase reporter system has been described 20, 59, 62. To generate the reporter constructs pGL3‐hymyc1 and pGL3‐hymyc2, a 416‐bp and a 319‐bp segment encompassing nucleotides −369 to +47 or nucleotides −216 to +103 of the *Hydra myc1* or *myc2* promoter regions, respectively, were amplified by PCR from genomic DNA using the primer pairs 5′‐GGATCCGGTACCCCCAATTATTAAAATTGTGGTGTG‐3′/5′‐GGATCCAAGCTTAGACTGTAATTTAAATTCGCAGCC‐3′, or 5′‐GGATCCGGTACCCTTGGTGTTTTTTATGTAATG‐3′/5′GGATCCAAGCTTTAGATCGGCTTTCATAGATA‐3′, and inserted into the *Kpn*I and *Hind*III sites of the pGL3‐Basic vector (Promega, Vienna, Austria). To construct pGL3‐c‐myc, a 691‐bp fragment of the chicken c‐*myc* promoter (accession no. J00889) encompassing nucleotides −621 to +107 was amplified from CEF cDNA and inserted into pGL3‐Basic. This insert encompasses the transcription start site (+1) and four consensus Tcf binding sites (5′‐YCTTTGWW‐3′). To create pRc‐hyTcf‐HA and pRc‐hyCtnnb‐HA, the coding sequences of *Hydra tcf* and of β‐*catenin* (*ctnnb*) were amplified from *Hydra* cDNA as described 18 using the primer pairs 5′‐TCGGCGGCCGCATGCCCCAACTACCAACTTCAACAG‐3′/5′‐TCGTCTAGATTAAGCGTAATCTGGAACATCGTATGGGTATCTAGTTTCAATTGCCTGAAGCGT TGG‐3′ and 5′‐TCGGCGGCCGCATGATGGAGGATTCAACTGCTCAAATGAG‐3′/5′‐TCGTCTAGACTAAGCGTAATCTGGAACATCGTATGGGTACAAGTCAGGGTCAAACCAACCCTG‐3′. The 3′‐primers contain the coding sequence of the hemagglutinin epitope (HA) fused in frame to the 3′‐end of the *tcf* or *ctnnb* coding sequences to allow immunological detection of the ectopic proteins. The PCR products were digested with *Not*I/(blunt ended) and *Xba*I and inserted into the eukaryotic pRc/RSV expression vector (Thermo Fisher Scientific, Vienna, Austria) which had been opened with *Hind*III/(blunt ended)/*Xba*I. Calcium phosphate‐mediated DNA transfection, and luciferase assays were performed as described 20.

### Cells and retroviruses

Cultivation of CEF and of the methylcholanthrene‐transformed cell line QT6 and cell transformation assays were performed as described 18, 59, 61. The construct pRCAS‐ΔLEF1ΔCTNNB (pRCAS‐ΔN‐bΔN) encoding a truncated mouse LEF1/CTNNB fusion protein has been described 38. To construct pRCAS‐hyCtnnb‐HA, the *Hydra ctnnb* insert from pRc‐hyCtnnb‐HA was released with *Xba*I/*Not*I/(blunt ended) and inserted into the *Cla*I/(blunt ended) pRCAS (BP) vector 63.

### Protein expression, purification, and analysis

To construct pET11d‐hyTcf(1‐151) a DNA segment encoding amino acid residues 2‐151 from *Hydra* Tcf was amplified by PCR using the primers 5′‐CCCCAACTACCAACTTCA‐3′/5′‐AAGCTTGGATCCTTAAGCTCCATATGGCAAAGG‐3′ and the plasmid pGEX6p3‐hyTcf 28 as a template. The PCR product was digested with *Bam*HI and inserted into pET11d vector yielding the construct pET11d‐hyTcf(1‐151) with the initiating methionine codon derived from the vector. The encoded *Hydra* Tcf(1‐151) protein (151 amino acids; *M*_r_ = 17 146; pI = 4.13) represents a truncated version of the 418‐amino acid full‐length *Hydra* Tcf protein. DNA of the construct pET11d‐hyTcf(1‐151) was transformed into *E. coli* strain BL21 (DE3) CodonPlus‐RIL (Stratagene/Agilent, Vienna, Austria). To express recombinant hyTcf(1‐151) protein, bacteria from a single colony were grown overnight at 37 °C with shaking at 220 r.p.m. in 10 mL of LB medium containing 100 μg·mL^−1^ ampicillin and 25 μg·mL^−1^ chloramphenicol. The bacteria were transferred into 400 mL LB medium containing 100 μg·mL^−1^ ampicillin, and grown at 37 °C with shaking at 220 r.p.m. to an optical density of 0.5 (600 nm). To induce recombinant protein expression, isopropyl‐β‐d‐thiogalactopyranoside was added to a final concentration of 1 mm and bacteria were incubated as above for 3 h. The bacteria were pelleted and resuspended in 25 mL of buffer A (20 mm Tris HCl pH7.5, 80 mm NaCl, 1 mm EDTA, 1 mm DTT, 1 mm PMSF), and then lysed at 1300 psi using a French Press. *DNAse* I was added to a final concentration of 5 μg·mL^−1^, and the lysate was incubated at 4 °C for 30 min. The sample was centrifuged at 18 000 ***g*** for 20 min at 4 °C. Ammonium sulfate was added to the clarified supernatant at 40% (w/v) saturation, and the solution was stirred on ice for 30 min. The precipitated proteins were pelleted by centrifugation at 11 000 ***g*** for 30 min at 4 °C. The pellet was dissolved in 10 mL of buffer A and dialyzed for 36 h at 4 °C against 2.5 L of buffer A, centrifuged at 18 000 ***g*** for 20 min, and then loaded onto a Mono Q anion exchange column using an automated liquid chromatography system (ÄKTA purifier; GE Healthcare). Chromatography was carried out with a linear gradient from 0 to 1 m NaCl in buffer A at a flow rate of 1 mL·min^−1^. Fractions containing hy Tcf(1‐151) protein were combined and applied onto a Superdex‐200 gel filtration column (GE Healthcare, Vienna, Austria) equilibrated with buffer A, and then eluted with the same buffer at a flow rate of 0.5 mL·min^−1^. hy Tcf(1‐151) containing fractions were pooled, dialyzed against PBS, and stored at −80 °C. The final yield of purified hy Tcf(1‐151) from the 400‐mL bacteria culture was approximately 500 μg. For electrospray ionization mass spectrometry (ESI‐MS), hy Tcf(1‐151) was desalted using Vivaspin 500 PES centrifugal concentrators (MWCO 5000) (Sartorius, Vienna, Austria). Centrifugal concentration and addition of 100 mm ammonium acetate in H_2_O (18 MΩ) to the supernatant was repeated six times, followed by six cycles of concentration and dilution with H_2_O (18 MΩ). The final protein concentration of the ESI solution (H_2_O : CH_3_OH vol/vol) was 1 μm. For ESI‐MS analysis in positive and negative ion mode, 1% vol/vol acetic acid and 0.1% 1,8‐diazabicyclo[5.4.0]undec‐7‐en (DBU) were added to aliquots of the ESI solution, respectively. SDS/PAGE and immunoblotting were done as described 18, 59. To prepare cell extracts from whole Hydra, ~ 10 animals were dissolved in 200 μL of RIPA buffer 59 and the extract clarified by centrifugation at 20 000 ***g*** for 1 h at 4 °C. The yield of total protein per animal was ~ 20 μg.

A polyclonal rabbit antiserum directed against the purified recombinant hyTcf(1‐151) protein was generated as described previously using 3 × 100 μg recombinant protein as immunogen 18, 20. The antiserum directed against *Hydra* Max has been described 18. The monoclonal mouse antibodies anti‐α‐tubulin, anti‐GFP, and anti‐HA were purchased from Sigma‐Aldrich, Vienna, Austria (T5168), Roche, Vienna, Austria (11 814 460 001), and Covance, Vienna, Austria (MMS‐101P), respectively. The polyclonal rabbit antibody directed against mouse anti‐β‐Catenin was purchased from Sigma‐Aldrich, Vienna, Austria (C2206).

*Hydra* Myc1‐ and Myc2‐specific antisera 18, 20 were subjected to immunoglobulin G (IgG) purification. To each 20 mL antiserum, an equal volume of saturated ammonium sulfate solution was added dropwise resulting in a final concentration of 50% (w/v), and stirred on ice for 3 h. After centrifugation at 3000 ***g*** for 20 min (4 °C), the pellets were washed twice in 50% (w/v) ammonium sulfate (20 mL), and centrifuged as above. Pellets were dissolved in 4 mL of PBS, and dialyzed against 20 mm sodium phosphate pH 7.0 for 48 h. Prior to protein G affinity chromatography, samples were clarified by centrifugation at 13 000 ***g*** for 30 min (4 °C), and then loaded onto a 5‐mL HiTrapG column (GE Healthcare, Vienna, Austria) equilibrated with 20 mm sodium phosphate pH 7.0. Bound IgG were eluted with 0.1 m glycine pH 2.7, and 1‐mL fractions immediately neutralized with 200 μL of 1 m Tris‐HCl pH 9.0. IgG fractions were pooled resulting in final volumes of 8 mL each, and dialyzed against 20 mm sodium phosphate pH 7.0 for 48 h. The IgG preparations were quantified (anti‐hy Myc1 540 μg·mL^−1^; anti‐hy Myc2 1080 μg·mL^−1^), and stored in aliquots at −80 °C. For immunoblotting, 1 : 50 dilutions of IgG‐purified antisera were applied.

## Conflicts of interest

The authors declare no conflict of interest.

## Author contributions

MH, KBi, and BH planned experiments, MH, SGl, SGu, AR, KP, and KBr performed experiments, MH, KBr, KBi, and BH analyzed data, MH and BH wrote the paper, with contributions from KBi.
